# Discrimination-related health inequality and role of social capital among marriage migrant women in South Korea

**DOI:** 10.1186/s12939-016-0464-z

**Published:** 2016-10-26

**Authors:** Chang-O Kim

**Affiliations:** Department of Social Welfare, Seoul National University, Gwanak Street 1, Gwank-gu, 151-746 Seoul, Republic of Korea

**Keywords:** Discrimination, Racism, Health inequality, Resilience, Linking social capital, Total differential decomposition, Power resource theory, South Korea

## Abstract

**Background:**

This study aimed to evaluate whether social capital could alleviate health inequality against racial discrimination and identify the critical nature of social capital that generates health inequality differences within the social context of South Korea.

**Methods:**

Using the data of the 2009 National Survey of Multicultural Families, a nationally representative sample in which 40,430 foreign wives participated, the concentration index (CI) was used to measure the discrimination-related inequalities in self-rated health and was decomposed into contributing factors.

**Results:**

The results showed a significant concentration of poor self-rated health unfavorable to foreign wives who were highly discriminated (CI 0.023, standard error [SE] 0.001, *p* < .001). However, when the CIs were assessed among the subgroups of different social capital, no discrimination-related inequality in health was observed among the group of linking social capital (CI 0.008, SE 0.008, *p* .332). The total differential decomposition method showed two major factors that generate differences in health inequality between the groups of non-linking and linking social capital: protest against discrimination (35.8 %); experiences of discrimination (28.3 %).

**Conclusions:**

The present results indicated that linking social capital can be a useful resource of health resilience factor that equalizes discrimination-related health inequality among marriage migrant women in South Korea. This study provides additional evidence that social capital needs to be placed in its political context.

## Background

Regarding the perspective of social determinants of health, racial discrimination is one of the important structural factors that produces health inequalities along racial and ethnic lines. There is growing evidence that perceived discrimination is associated with lower levels of physical and mental health, poor access to quality healthcare, and certain health behaviors across several immigrant groups [1]. These effects may be cumulative, rather than transient, because individual experiences of day-to-day discrimination are rooted in the structural process of ‘othering,’ which locates individuals differentially within the ethnoracial hierarchy [2].

Despite the existence of discrimination-related inequality, it has been posited that health resilience factors exhibit better health outcomes than would be expected, given the setting of adversity or risk [3, 4]. One idea promoted in the literature is that social capital could be an alternative resource to help the disadvantaged and their community get ahead [5, 6]. According to previous studies, those who participated in the social group scored higher on self-rated health surveys and displayed greater functional capacity [7]. However, the association between social capital and its effect on health inequality—not on health status using the conditional-mean model (i.e., a measure of central tendency)—has not been well-defined among marriage migrant women [8]. Moreover, although income, expenditure, or proxy measure of wealth were widely used to measure the health inequality index in the previous studies [9], there is a paucity of empirical studies that address the discrimination-related health inequality index using the total inequality approach.

Is there inequality in health according to the rank of discrimination experiences among marriage migrant women in South Korea? If so, what is the role of social capital? Can social capital operate as a resilience factor for alleviating health inequality? In this study, I aimed to examine the critical nature of social capital and the underlying resources embedded in the network, using the decomposition method of the concentration index. Before outlining and testing the analytic model, I discuss the historical and social context of marriage migrant women in South Korea.

### Marriage migrant women’s health in South Korea

Korean society has traditionally had a deep-rooted sense of ethnic nationalism that emphasized the principle of ‘one race’ and kinship [10]. However, over the last two decades, South Korea has become more ethnically diverse. There was a rapid increase in international marriages, which accounted for 7.6 % of all marriages in 2014, a seven-fold increase from 1.2 % in 1990 [11].

Since then, the social identity and immigrant status of marriage migrants in South Korea have been differently constructed according to gender because the majority of the foreign wives came from less-developed countries (e.g., China, Vietnam, and the Philippines); in contrast, most of the foreign husbands had citizenship from well-developed countries (e.g., USA, Canada, and England). First, this difference is caused by an increased demand of foreign wives in the marriage market because of the widening male/female sex ratio in South Korea, especially in the rural and inner city areas; second, this difference is attributed to the government policies that promote childbirth with a new paradigm of multiculturalism. Consequently, international marriage arranged by a commercial marriage broker and the cases of insufficient wedding plans were drastically increased since the early 2000s. For example, the foreign spouses could not easily adapt to their partner’s culture and language, and stress-related health outcomes, such as depression and sickness behavior, have been highly reported in the early stage of immigration [12, 13].

However, discrimination and negative stereotype attached to foreign wives’ daily lives might affect health status more profoundly and consistently than cultural factors in the perspective of intersectionality theory [14, 15]. Contrary to the articulating gender, race, and working class of the husband’s origin as distinct social categories, intersectionality postulates that these systems of oppression are mutually constituted and work together to produce inequality [2, 16]. Within the social context of South Korea, marriage migrant women experienced various types of discrimination that have a negative influence on their subjective health [10, 12, 17, 18]. Typically, negative social identity formulated by discrimination has led foreign wives to social isolation not only from the Korean society but also from their own ethnic group [10].

How does social capital work within the racialized structure that produces and reproduces marginalization and exclusion in South Korea? In the field of health inequality research, an abundance of evidence indicates that social capital moderates or mediates the relationship between socioeconomic inequalities and health [8]. However, when the variables of race, gender, and discrimination are integrated into this relationship, it is unclear whether social capital could alleviate health inequality and could be regarded as a useful resource for foreign wives. Moreover, when the nature of social capital is deconstructed into *relational* and *material* elements, as Portes [19] had suggested, or into *bonding*, *bridging*, and *linking* components, as Szreter and Woolcock [5] had proposed, additional complexities emerge, thereby making it difficult to answer the questions that must be addressed. This article aimed to fill this gap of existing studies by analyzing the data of marriage migrant women’s health in South Korea.

## Methods

### Data source and study participants

The study participants were derived from an entire population of 131,702 marriage migrants residing in South Korea in 2009. Based on the Support for Multicultural Families Act, enacted in March 2008, the Korean government decided to conduct an official census of marriage migrants to investigate and obtained detailed information on household demographics, family relationship, health status, and social well-being. Approximately 3000 census takers, who were managed by 160 supervisors, visited the migrants’ residence and obtained self-reported responses using the ethnically competent questionnaire. Of 131,702 marriage migrants in the government database, which were initially investigated by the Ministry of Public Administration and Security, a total of 73,669 (55.9 %) marriage migrants agreed to participate in the 2009 National Survey of Multicultural Families. Because this study focused on discrimination-related health inequality of foreign wives, 4275 foreign husbands and 22,291 marriage migrant women who had already acquired Korean citizenship were excluded from the study. An additional 6664 people were excluded because of missing data (855 for self-reported health, 1111 for discrimination experiences, 4698 for length of residence, 9 for age); the resulting study population consisted of 40,430 (30.7 %) foreign wives. The nonresponse weight was applied in the analyses to compensate for the differential response rates by country of origin and residential district. The study protocol was reviewed and approved by the Institutional Review Board of Seoul National University (E1604/001-001).

### Variables

The health outcome, poor self-rated health (SRH), was measured using the following questions: “How would you rate your overall health? Would you say it is: very good (1), good (2), fair (3), bad (4), very bad (5)?” The SRH status has proven to be an independent strong predictor of overall mortality [20]. Because dichotomizing the categorical health indicators is potentially unreliable when analyzing health inequalities [21], it is postulated that SRH is ordinal variable with the uniform intervals between the categories. Thus, in this study, poor SRH could be interpreted as ill-health score, ranging from 1 to 5.

Self-reported experiences of discrimination was measured using a modified version of the Experiences of Discrimination questionnaire [22]. The respondents were asked whether they had “ever experienced discrimination in any of the following five situations because you are a foreigner while living in Korea”: 1) in the street or in the neighborhood; 2) at stores, restaurants, banks, etc.; 3) in public offices (district offices, police station, etc.); 4) by landlords or real estate agents; and 5) at work. For each question, the options for the response were as follows: very strong (4); quite strong (3); not so strong (2); little (1); and no discrimination or not applicable (0). It is argued that when collecting the data, the questions should be direct and address the multiple facets of discrimination by focusing on the distinct types of unfair treatment in particular situations and locations [23]. The magnitude of discrimination were also assessed [24]. In this study, a summary discrimination score, ranging from 0 to 20, was calculated by summing all five questions. The Cronbach’s α in the current study was 0.90.

Social capital was defined as the ability to secure benefits through membership in networks and other social structures [19] and was measured using associational membership. The respondents were asked whether they participated in six types of associations during the preceding year: home country friends’ meetings; school parents’ meeting; community association; civil society organization; labor union; and political party. The operationalization of the types of social capital was achieved in two ways. First, bonding and bridging social capital was distinguished by perceived similarity to other members of a group with respect to race/ethnicity [25]. In this study, the foreign wives who replied that they participated only in the ‘home country friends’ meetings’ were categorized as being involved with bonding social capital. Second, bridging and linking social capital was distinguished by structural similarity with respect to ‘explicit, formal, or institutionalized power or authority.’ [5] From this perspective, linking social capital (vertical tie), rather than bridging social capital (horizontal tie), provided access to the valued resources between and among dissimilar groups [6]. In this study, foreign wives who replied that they participated in either civil society organization, labor union, or political party were categorized as being involved with linking social capital. Although few studies have measured this distinction [26–30], it could emphasize the material and political aspects of social capital, which has often been neglected in public health research [31–33].

The demographic and socioeconomic variables in this analysis included age, length of residence in South Korea, country of origin, household income, marital status, educational level, and economic activity. The following three well-known structural factors that affect both health inequality and discrimination were selected for the decomposition analysis: 1) language proficiency; 2) change of subjective social position; and 3) protest against discrimination. Language proficiency was measured using the Korean Language Literacy Scale, a self-assessed five-point scale to evaluate each individual’s ability to speak, read, and write in the Korean language, creating a global score that ranged from 1 (worst proficiency) to 15 (best proficiency). Cronbach’s α of the scale was 0.94 in the present study. The subjective social position refers to an individual’s perceived social position in a social hierarchy [34]. The respondents were asked about the following questions and were instructed to place an “X” on the ladder with rungs that were assigned numbers ranging from 0 to 10, whereby 0 indicated the bottom and 10 indicated the top of the social position: “What do you think is your family’s socioeconomic status 1) in Korea? and 2) in your home country?” By subtracting the scores obtained for “in your home country” from the scores obtained for “in Korea,” the change in the subjective social position score, ranging from −10 to 10, was calculated. Protest against discrimination was measured using this question: “Regarding your discrimination experiences, have you requested a correction?” The participants answered this question with either yes or no/not applicable. According to the literature, the problem-focused coping style (i.e., confrontation) has been found to be more effective in reducing the mental and physical health impact of perceived discrimination compared with the emotional-focused coping style (e.g., screaming or crying, taking the problem out on someone else, and doing something to forget) [23, 24].

### Statistical analysis

One-way analysis of variance and post-hoc multiple comparison test with Bonferroni correction was used to compare between-group differences of poor SRH scores. The concentration curve (CC) and the concentration index (CI) were used to measure the total inequalities in SRH among marriage migrant women. Conventionally, the CC plots the cumulative percentage of the health variable (y-axis) against the cumulative percentage of the population, ranked by living standards, commencing with the poorest and concluding with the richest (x-axis), to assess income- or wealth-related inequality in health. In this study, the CC plots the shares of poor SRH against the rank of the variable of discrimination to identify the degree to which discrimination contributes to inequality. Then, two or more CCs of marriage migrant women were plotted differently according to the type of social capital. To evaluate whether there is Lorenz dominance between the two curves, a multiple comparison test using 19 rank points was performed [9, 35].

The CI is defined as twice the area between the CC and the 45° line (i.e., line of equality). So, if everyone has exactly the same value of SRH, irrespective of his/her experiences of discrimination, the CC will be on a line of equality and the CI will be calculated as zero. If, in contrast, the poor SRH variable has higher values among the individuals who experienced severe discrimination, the CC will lie below the line of equality and the CI will approach 1 because of the cumulative percentage of the sample (x-axis) commencing with the persons who did not perceive any discrimination. In this study, the CI was estimated using the convenient regression method as Kakwani et al. [36] had proposed. Using this method, both the standardized and representative CIs could be easily acquired by adding the demographic variables and applying the sampling weight in the regression formula.

Wagstaff, van Doorslaer, and Watanabe [37] demonstrated that if the health variable *y* can be explained linearly using a set of *k* determinants *x*_*k*_ through a regression model

1 $$ {y}_i=\upalpha +{\sum}_k{\beta}_k{x}_{ki}+{\varepsilon}_i, $$

then, the CI for the health variable can be decomposed into contributions from the determinants as follows:

2 $$ \mathrm{C}\mathrm{I}={\sum}_k\left(\frac{\beta_k{\overline{x}}_k}{\mu}\right)\mathrm{C}{\mathrm{I}}_k+\frac{\mathrm{GC}{\mathrm{I}}_{\varepsilon }}{\mu }, $$

where *β*_*k*_, ${\overline{x}}_k$, and CI_*k*_ are the coefficient, mean, and CI of *x*_*k*_, respectively; *μ* is the mean of y; and GCI_*ε*_/*μ* is a residual that should approach zero for a well-specified model. They also proposed that the differences in the CI between the two groups (i.e., dCI) can be approximately decomposed into a weighted sum of the differences in *β*_*k*_, ${\overline{x}}_k$, and CI_*k*_, as shown below:

3 $$ \mathrm{d}\mathrm{C}\mathrm{I}={\sum}_k\frac{{\overline{x}}_k}{\mu}\left(\mathrm{C}{\mathrm{I}}_k-\mathrm{C}\mathrm{I}\right)\mathrm{d}{\beta}_k+{\sum}_k\frac{\beta_k}{\mu}\left(\mathrm{C}{\mathrm{I}}_k-\mathrm{C}\mathrm{I}\right)\mathrm{d}{\overline{x}}_k+{\sum}_k\frac{\beta_k{\overline{x}}_k}{\mu}\mathrm{d}\mathrm{C}{\mathrm{I}}_k+\mathrm{d}\frac{\mathrm{GC}{\mathrm{I}}_{\varepsilon }}{\mu }-\frac{\mathrm{CI}}{\mu}\mathrm{d}\upalpha . $$

This total differential decomposition method allows us to explain the differences in health inequality among the groups of different social capital. The differences in discrimination-related health inequality (e.g., linking social capital vs. non-linking social capital) can be attributed to the differences in the determinant’s impact on health or the differences in the means of the various determinants, or the differences in the degree of inequality in its determinants. Although such analyses are purely descriptive, it is likely to identify the factors that generate health inequality, if the data are sufficient to allow the estimation of causal effects [9].

## Results

The basic characteristics of the study participants are described in Table 1. In the sample, 36.9 and 26.9 % of female marriage migrants were classified as bonding and bridging social capital, respectively, and 34.0 % reported that they did not participate in any association. Only 2.2 % of marriage migrant women were active members of either a civil society organization (*n* = 779), labor union (*n* = 557), or political party (*n* = 361). The participants with linking social capital were more likely to experience severe discrimination and were in poorer health. They were also more likely to report being older, having a longer length of residence in Korea, having a better command of the Korean language, coming from a country other than China or Vietnam, having a Bachelor’s or higher degree of education, having an occupation, being perceived as a decline in social position after immigration, and using more active coping skills, such as protest against discrimination.

**Table 1:** General characteristics of the study participants in addition to types of social capital

	Total (*n* = 40430)	Social capital				
		None (*n* = 12553)	Bonding (*n* = 14518)	Bridging (*n* = 12359)	Linking (*n* = 1000)	*p* ^*^
Weighted proportion, %	100.0	34.0	36.9	26.9	2.2	
Self-rated poor health, score	2.33 (0.92)	2.36 (0.94)	2.28 (0.90)	2.35 (0.90)	2.50 (0.92)	<.001
Experiences of discrimination, score	2.28 (3.90)	2.18 (3.86)	2.36 (3.90)	2.22 (3.86)	3.44 (4.72)	<.001
Demographics						
Age, year	32.0 (9.4)	33.9 (10.1)	30.9 (8.7)	30.9 (8.8)	35.1 (9.7)	<.001
Length of residence, month	42.3 (39.9)	37.6 (34.7)	37.5 (32.9)	51.9 (47.8)	80.4 (68.2)	<.001
Country of origin, %						
China	49.4	67.9	49.4	27.6	28.0	<.001
Vietnam	26.4	18.0	27.2	37.1	13.8	
Others	24.2	14.1	23.4	35.3	58.2	
Household income, %						
< 1 million KRW (US $935)	18.3	19.9	15.3	20.2	20.2	<.001
1–2 million KRW	37.6	38.4	39.2	34.8	35.8	
≥ 2 million KRW (US $1,870)	27.5	24.8	30.5	26.7	27.7	
Missing	16.6	16.9	15.0	18.3	16.3	
Marital status, %						
Married	93.9	92.4	94.3	95.4	92.6	<.001
Separated/divorced/widowed	4.1	5.2	4.0	2.9	5.0	
Missing	2.0	2.4	1.7	1.7	2.4	
Educational level, %						
Less than Bachelor’s degree	77.7	82.9	77.5	73.1	56.6	<.001
Bachelor’s degree or higher	21.5	16.2	21.8	26.2	42.6	
Missing	0.8	0.9	0.7	0.7	0.8	
Economic activity, %						
Unemployed (homemaker)	67.0	63.5	67.6	71.2	60.6	<.001
Employed	31.8	35.0	31.5	27.8	37.6	
Missing	1.1	1.5	0.9	1.0	1.8	
Additional variables						
Language proficiency, score	8.3 (3.4)	8.1 (3.7)	8.3 (3.2)	8.5 (3.1)	9.0 (3.1)	<.001
Change of social position, score	−0.5 (2.2)	−0.6 (2.3)	−0.4 (2.2)	−0.3 (2.1)	−0.7 (2.3)	<.001
Protest against discrimination, %	9.2	8.5	9.8	9.0	13.3	<.001

*Source:* 2009 National Survey of Multicultural Families, Republic of Korea

*Note:* The data are presented as weighted proportion or weighted mean (standard deviation) according to the variable characteristics. The nonresponse weight provided from the data source was applied

^*^ *p* values were calculated between the group values from the chi-squared test (without applying the sample weight) except for six variables of the following; *p* values for experiences of discrimination and length of residence were calculated using the Kruskal-Wallis test; *p* values for self-rated poor health, age, language proficiency, and change of social position were calculated using the one-way ANOVA (F-test)

**Fig. 1:**
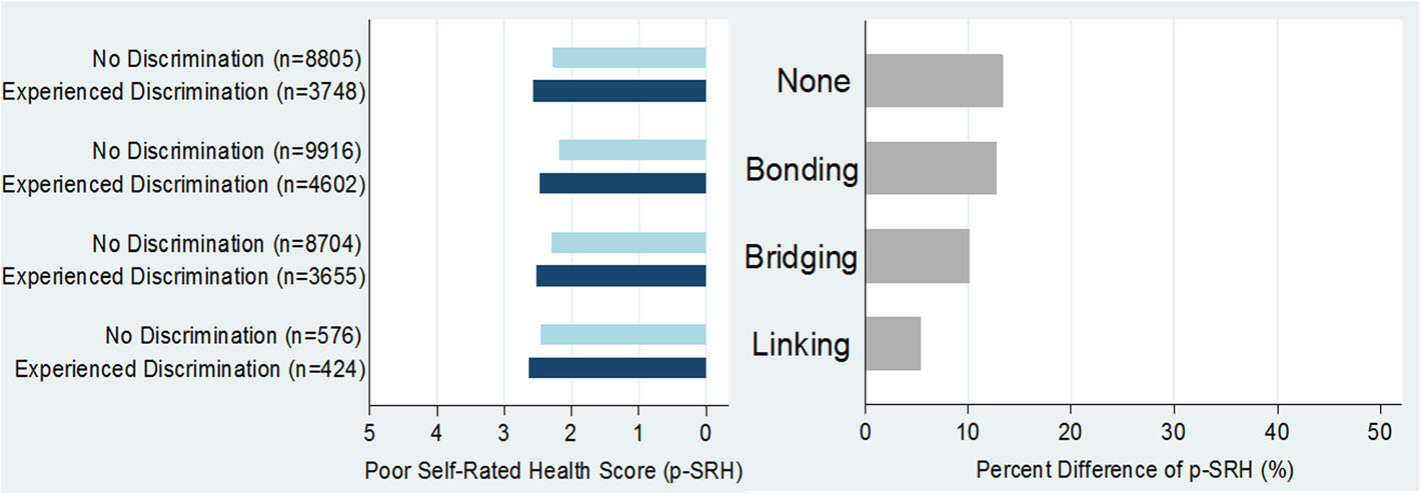
Percent differences of poor self-rated health among marriage migrant women between the subgroups of discrimination (D) and non-discrimination (ND) according to the types of social capital. (Note: Percent differences were calculated by the following equation: {(D score – ND score) / ND score}*100. ‘ND’ if a summary discrimination score is zero; else ‘D’.)

Table 2 shows the group differences of poor SRH score between social capital categories according to the binary variable of discrimination experience among marriage migrant women in South Korea. Among the subgroup of participants who said they did not experience any discrimination (*n* = 28001), there showed marked group differences of SRH across the types of social capital (F-test *p* < .001; post-hoc test *p* < .001 [excepting none vs. bridging *p* 1.000]). However, these differences were considerably decreased among the marriage migrant women who reported they had experienced racial discrimination (*n* = 12429; F-test *p* < .001; post-hoc test *p* ≥ .140 [excepting none vs. bonding *p* < .001]). It implies that health-enhancing effect of social capital (e.g., bonding social capital) may be attenuated in the circumstances of racial discrimination. Figure 1 shows the results of percent differences of poor SRH score between the groups of discrimination experiences (i.e., experience of discrimination ‘no’ vs. ‘yes’) according to the types of social capital. Among the subgroup, there are four ranks of between-group differences: none (13.2 %), bonding (12.8), bridging (10.1 %), and linking social capital (5.3 %) from highest to lowest.

**Table 2:** Group differences of poor self-rated health score among types of social capital according to the characteristics of discrimination experiences

	Experience of discrimination					
	No (*n* = 28001)			Yes (*n* = 12429)		
	%	mean (SD)	*p*	%	mean (SD)	*p*
Self-rated poor health, score						
No social capital	34.5	2.26 (0.92)	<.001^a^	33.0	2.56 (0.97)	<.001^a^
Bonding social capital	36.3	2.19 (0.88)		38.2	2.47 (0.93)	
Bridging social capital	27.4	2.28 (0.89)		26.0	2.51 (0.90)	
Linking social capital	1.8	2.45 (0.90)		2.9	2.58 (0.93)	
Group Difference (B – A)						
None (A) vs. Bonding (B)		−0.08	<.001^b^		−0.09	<.001^b^
None (A) vs. Bridging (B)		0.01	1.000^b^		−0.05	.140^b^
None (A) vs. Linking (B)		0.19	<.001^b^		0.01	1.000^b^
Bonding (A) vs. Bridging (B)		0.09	<.001^b^		0.04	.201^b^
Bonding (A) vs. Linking (B)		0.26	<.001^b^		0.11	.142^b^
Bridging (A) vs. Linking (B)		0.17	<.001^b^		0.06	1.000^b^

*Abbreviations*: *SD* standard deviation

*Source:* 2009 National Survey of Multicultural Families, Republic of Korea

*Note:* Experiences of discrimination is a dichotomous variable (‘No’ if a summary discrimination score is zero; else ‘Yes’). The data are presented as weighted proportion or weighted mean (standard deviation). The nonresponse weight provided from the data source was applied

^a^ *p* values were calculated using the one-way ANOVA (F-test)

^b^ *p* values were calculated using post-hoc multiple comparison test with Bonferroni correction

**Fig. 2:**
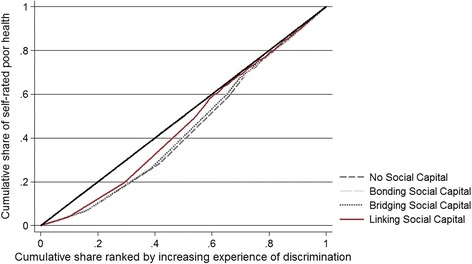
Concentration curves for discrimination-related health inequality according to the types of social capital among marriage migrant women in South Korea

The CCs for the discrimination-related health inequality according to the types of social capital are shown in Fig. 2. The curves provide the unadjusted results of the cumulative share of poor SRH against the cumulative share ranked by experiences of discrimination among the groups of different social capital, illustrating that ill health is more equally distributed in the group of linking social capital. The multiple comparison test showed that there is at least one quantile point at which the curve of linking social capital lies significantly above the curves of bonding or bridging social capital and no quantile point at which the curve of bonding or bridging social capital lies above the curve of linking social capital (Table 3). Then, it is concluded that linking social capital dominates both bonding and bridging social capital. In contrast, there was no evidence of Lorenz dominance between the CCs of linking and no social capital (Table 3).

**Table 3:** Dominance test between concentration curves according to the types of social capital

Data 1	Data 2	Results	Sig. level	# points	Rule
None	Bonding SC	‘None’ dominates ‘Bonding SC’	5 %	19	mca
None	Bridging SC	curves cross	5 %	19	mca
None	Linking SC	non-dominance	5 %	19	mca
Bonding SC	Bridging SC	curves cross	5 %	19	mca
Bonding SC	Linking SC	‘Linking SC’ dominates ‘Bonding SC’	5 %	19	mca
Bridging SC	Linking SC	‘Linking SC’ dominates ‘Bridging SC’	5 %	19	mca

*Abbreviations*: *SC* social capital, *mca* multiple comparison approach, *Sig.* significance, *#* number

*Source:* 2009 National Survey of Multicultural Families, Republic of Korea

*Note:* The nonresponse weight provided from the data source was applied. Dominance test was conducted by the user-developed program *dominance* [9]

Table 4 presents the results of the CI estimation among the groups of different types of social capital using a different adjusted regression model. The discrimination-related CIs among the total sample and the subsample of no social capital, bonding social capital, and bridging social capital were 0.023, 0.022, 0.023, 0.024, respectively, indicating that poor SRH was generally more concentrated in marriage migrant women who were highly discriminated against (CI 0.022-0.024, SE 0.001–0.003, *p* < .001). However, the CI among the subgroup of linking social capital had markedly declined to the value of 0.008 and was unable to reject the null hypothesis that the CI is equal to zero (unadjusted model: CI 0.008, SE 0.008, *p* .332). This situation implies that linking social capital may possess a health-equalizing effect against racial discrimination. This result did not change fundamentally after controlling for the confounding effect of demographic and socioeconomic factors (fully adjusted model: CI 0.007, SE 0.012, *p* .442). For comparison purposes, the regression models of income-related inequality were also fitted, which reflects the relationship between the poor SRH and the rank variable of household income. The CI values did not differ much among the subgroup of the different social capital, when health inequality were assessed using the popular variable of economic living standards (unadjusted model: CI −0.030 to −0.023, SE 0.002–0.008, *p* ≤ .003; and fully adjusted model: CI −0.027 to −0.025, SE 0.002–0.014, *p* ≤ .014).

**Table 4:** Concentration indexes for inequality in self-rated poor health among marriage migrant women in South Korea

	*n*	Unadjusted model		Fully adjusted model	
		CI (SE)	*p*	CI (SE)	*p*
Discrimination-related inequality					
Total sample	40430	0.023 (0.001)	<.001	0.018 (0.002)	<.001
Subsample					
No social capital (A)	12553	0.022 (0.003)	<.001	0.018 (0.005)	<.001
Bonding social capital (B)	14518	0.023 (0.002)	<.001	0.017 (0.004)	<.001
Bridging social capital (C)	12359	0.024 (0.002)	<.001	0.018 (0.004)	<.001
Non-linking social capital (A + B + C)	39430	0.023 (0.001)	<.001	0.018 (0.002)	<.001
Linking social capital (D)	1000	0.008 (0.008)	.332	0.007 (0.012)	.422
Income-related inequality					
Total sample	33108	−0.026 (0.001)	<.001	−0.027 (0.002)	<.001
Subsample					
No social capital (A)	10264	−0.030 (0.003)	<.001	−0.027 (0.005)	<.001
Bonding social capital (B)	12115	−0.023 (0.002)	<.001	−0.026 (0.004)	<.001
Bridging social capital (C)	9901	−0.024 (0.002)	<.001	−0.026 (0.004)	<.001
Non-linking social capital (A + B + C)	32280	−0.026 (0.002)	<.001	−0.027 (0.002)	<.001
Linking social capital (D)	828	−0.025 (0.008)	.003	−0.025 (0.014)	.014

*Abbreviations*: *n* sample size, *CI* concentration index, *SE* standard error

*Source:* 2009 National Survey of Multicultural Families, Republic of Korea

*Note:* CIs, SEs, and *p* values were estimated using the convenient regression: $2{\sigma}_r^2\left(\frac{h_i}{\mu}\right)=\upalpha +\upbeta {r}_i+{\sum}_j{\delta}_j{x}_{ji}+{\varepsilon}_i,$

where *σ*_*r*_^2^ and *h*_*i*_/*μ* are the variance of the fractional rank (*r*_*i*_) and share of the poor self-rated health, respectively. *r*_*i*_ is selected as the rank of discrimination experiences to estimate the discrimination-related CI; in contrast, *r*_*i*_ is selected as the rank of household income to estimate the income-related CI. *x*_*j*_ represents the confounding variables listed below to estimate indirectly standardized CI. For the discrimination-related inequality model, variables include age, length of residence, country of origin, household income, marital status, educational level, economic activity, language proficiency, change of subjective social position, and protest against discrimination. For the income-related inequality model, the variable of experiences of discrimination (dichotomous variable) was additionally inserted and the variable of household income was excluded from the model. The nonresponse weight provided from the data source was applied

Table 5 provides the results of the decomposition analysis for the survey data according to the two distinct types of social capital, linking and non-linking social capital. The first column under the heading ‘*β* (SE)’ shows the results of the regression coefficients to describe the influence of each determinant on poor SRH. For example, the experiences of discrimination was positively associated with ill health among the group of non-linking social capital (*β* 0.218, SE 0.12, *p* < .001); in contrast, this effect was markedly decreased among the group of linking social capital (*β* 0.163, SE 0.07, *p* .018). Additionally, a qualitatively different effect of ‘protest’ on health was observed among the group of different social capital. Although these results did not reach 10 % of statistical significance level, protest against discrimination was positively associated with ill health among the group of non-linking social capital (*β* 0.033, SE 0.02, *p* .132); in contrast, there was a negative association between protest and ill health among the group of linking social capital (*β* -0.122, SE 0.10, *p* .238).

**Table 5:** Decomposition of discrimination-related health inequalities between non-linking vs. linking social capital among marriage migrant women in South Korea

	Non-linking social capital (*n* = 39430)						Linking social capital (*n* = 1000)					
	*β* (SE)	$\overline{x}$	$\beta \overline{x}/\mu$	CI	Total	(%)	*β* (SE)	$\overline{x}$	$\beta \overline{x}/\mu$	CI	Total	(%)
Experiences of discrimination^a^	0.218 (0.12)^‡^	0.32	0.030	0.656	0.020	85.2	0.163 (0.07)^*^	0.43	0.028	0.549	0.015	194.9
Demographics												
Age	0.016 (0.00)^‡^	31.9	0.217	−0.004	−0.001	−3.3	0.021 (0.01)^‡^	35.1	0.300	0.010	0.003	39.1
Length of residence (log)	0.097 (0.01)^‡^	3.40	0.142	0.014	0.002	8.4	0.052 (0.05)	4.00	0.083	0.023	0.002	23.7
Country of origin: China	0.071 (0.01)^‡^	0.50	0.015	−0.004	−0.000	−0.3	−0.084 (0.09)	0.28	−0.009	−0.000	0.000	0.0
Country of origin: Vietnam	0.102 (0.01)^‡^	0.27	0.012	−0.008	−0.000	−0.4	−0.070 (0.11)	0.14	−0.004	−0.116	0.000	5.7
Income: <1 million KRW	0.284 (0.02)^‡^	0.18	0.022	0.052	0.001	5.0	0.262 (0.10)^†^	0.20	0.021	0.011	0.000	2.9
Income: 1–2 million KRW	0.146 (0.01)^‡^	0.38	0.024	0.010	0.000	1.0	0.173 (0.08)^*^	0.36	0.025	0.008	0.000	2.6
Income: Missing	0.248 (0.02)^‡^	0.17	0.018	−0.011	−0.000	−0.8	0.126 (0.10)	0.16	0.008	−0.101	−0.001	−10.5
Marital status: Separated	0.105 (0.03)^‡^	0.04	0.002	0.026	0.000	0.2	0.143 (0.19)	0.05	0.003	−0.044	−0.000	−1.6
Marital status: Missing	−0.145 (0.03)^‡^	0.02	−0.001	−0.054	0.000	0.3	0.098 (0.28)	0.02	0.001	−0.113	−0.000	−1.3
Education: ≥Bachelor’s degree	−0.077 (0.01)^‡^	0.21	−0.007	0.014	−0.000	−0.4	−0.087 (0.07)	0.43	−0.015	0.069	−0.001	−13.0
Education: Missing	0.033 (0.05)	0.01	0.000	−0.019	−0.000	−0.0	−0.033 (0.44)	0.01	−0.000	0.050	−0.000	−0.1
Economic activity: Employed	−0.042 (0.01)^†^	0.32	−0.006	0.046	−0.000	−1.1	0.026 (0.07)	0.38	0.004	0.043	0.000	2.1
Economic activity: Missing	−0.102 (0.04)^*^	0.01	−0.000	−0.055	0.000	0.1	−0.130 (0.30)	0.02	−0.001	−0.288	0.000	3.4
Additional variables												
Language proficiency	−0.022 (0.00)^‡^	8.29	−0.078	0.005	−0.000	−1.6	−0.030 (0.01)^†^	8.99	−0.106	0.014	−0.002	−19.2
Change of social position	−0.020 (0.00)^‡^	−0.46	0.004	0.204	0.001	3.4	−0.002 (0.02)	−0.73	0.001	0.130	0.000	0.9
Protest against discrimination	0.033 (0.02)	0.09	0.001	0.684	0.000	3.8	−0.122 (0.10)	0.13	−0.006	0.598	−0.004	−49.3
Residual					0.000	0.6					−0.006	−80.4
Total					0.023	100.0					0.008	100.0

*Abbreviations*: *β* regression coefficient, *SE* standard error, $\overline{x}$ mean of determinants, *μ* mean of poor self-rated health, *CI* concentration index

*Source:* 2009 National Survey of Multicultural Families, Republic of Korea

*Note:* β (SE) and CI of each determinant were estimated by Equations (1) and (2), respectively. The reference groups were listed as follows: Others (country of origin), ≥2 million KRW (income), Married (marital status), Less than Bachelor’s degree (education), Unemployed (economic activity). The nonresponse weight provided from the data source was applied

^a^Experiences of discrimination is a dichotomous variable (0 if a summary discrimination score is zero; else 1)

^*^ *p* < .05; ^†^ *p* < .01; ^‡^ *p* < .001 (two-tailed test)

By multiplying the values in the ‘$\beta \overline{x}/\mu$’ and ‘CI’ column, the contributions of each determinant toward the discrimination-related inequality were described in the fifth and sixth columns under the heading ‘Total (%).’ Among the group of non-linking social capital, the largest contribution to inequality came from the discrimination experiences (85.2 %), followed by length of residence (8.4 %). Similar patterns were observed in the group of linking social capital. However, the contribution of discrimination (194.9 %), age (39.1 %), and length of residence (23.7 %) became larger due to the increased contribution of protest against discrimination (−49.3 %) and the unexplained factors (−80.4 %) in the opposite direction.

Table 6 shows the results of total differential decomposition to explain the critical nature of linking social capital that produces the difference in discrimination-related health inequality. The total CI difference between the subgroups of non-linking and linking social capital was 0.0154. The last column under the heading ‘Total (%)’ shows that the main contributor of this difference was protest against discrimination (35.8 %) and experiences of discrimination (28.3 %), although there exist largely unobserved factors (45.5 %) yet to be explained. For protest against discrimination, the differences in the coefficients (0.0049) and the mean values (0.0012)—rather than protest inequality (−0.0006)—accounted for the bulk of the widening inequality between the two groups of social capital. In the case of experiences of discrimination, in contrast, the difference in coefficients (0.0052) and the uneven distribution of discrimination itself (0.0030) appear to be more important than the difference in the prevalence of discrimination (−0.0038).

**Table 6:** Decomposition of the difference in discrimination-related health inequalities between the non-linking vs. the linking social capital among marriage migrant women in South Korea

	Total differential decomposition					
	β’s	Mean of x’s	CIs	GC_ε_	Total	(%)
Experiences of discrimination^a^	0.0052	−0.0038	0.0030		0.0044	28.3
Demographics						
Age	−0.0002	−0.0001	−0.0041		−0.0044	−28.6
Length of residence (log)	0.0011	−0.0002	−0.0007		0.0002	1.0
Country of origin: China	−0.0001	−0.0001	−0.0000		−0.0000	−0.3
Country of origin: Vietnam	−0.0012	0.0004	−0.0004		−0.0011	−7.5
Income: <1 million KRW	0.0000	−0.0000	0.0009		0.0009	5.7
Income: 1–2 million KRW	0.0000	0.0000	0.0000		0.0001	0.3
Income: Missing	−0.0009	−0.0000	0.0007		−0.0001	−0.9
Marital status: Separated	0.0000	0.0000	0.0002		0.0003	1.7
Marital status: Missing	0.0003	0.0000	0.0001		0.0004	2.3
Education: ≥Bachelor’s degree	0.0001	0.0005	0.0008		0.0014	9.0
Education: Missing	0.0000	0.0000	0.0000		0.0000	0.1
Economic activity: Employed	−0.0004	−0.0000	0.0000		−0.0004	−2.3
Economic activity: Missing	−0.0001	−0.0001	−0.0002		−0.0004	−2.5
Additional variables						
Language proficiency	0.0002	0.0001	0.0010		0.0012	8.0
Change of social position	0.0006	−0.0000	0.0001		0.0007	4.2
Protest against discrimination	0.0049	0.0012	−0.0006		0.0055	35.8
Residual					0.0070	45.5
Total	0.0096	−0.0020	0.0008	0.0070	0.0154	100
Percent	62.3	−12.8	5.0	45.5	100	

*Abbreviations*: *β* regression coefficient, *CI* concentration index; *GC*_*ε*_ generalized concentration index for residual

*Source:* 2009 National Survey of Multicultural Families, Republic of Korea

*Note:* β’s, mean of x’s, CIs, and GC_ε_ were estimated in Equation (3). The reference groups were listed as follows: Others (country of origin), ≥2 million KRW (income), Married (marital status), Less than Bachelor’s degree (education), Unemployed (economic activity). The nonresponse weight provided from the data source was applied

^a^Experiences of discrimination is a dichotomous variable (0 if a summary discrimination score is zero; else 1)

## Discussion

This study is one of the first to suggest an association between the linking social capital and the health-equalizing effect against racial discrimination in South Korea. There exists a significant concentration of poor SRH unfavorable to foreign wives who are highly discriminated. However, when the CIs were assessed among the subgroups of different social capital, no discrimination-related inequality in health was observed among the group of linking social capital. The total differential decomposition method revealed the following two major factors that generate differences in health inequality between the groups of non-linking and linking social capital: 1) different prevalence and health impact of protest against discrimination; 2) different distribution and health impact of discrimination itself.

How plausible are these empirical results? How can we explain that participating in either a civil society organization, labor union, or a political party carries the embedded meaning of a health-equalizing effect among foreign wives? First, different prevalence and impact on health of protest against discrimination could be explained by the theory concerning the power resource of different populations. According to the thesis of Walter Korpi [38], who originated the power resources theory, the greater the difference in power resources between two actors, the lower is the motivation of the weaker actor to exercise power resource in relation to the stronger one (i.e., non-issues and non-decision-making). Because conflict requires that both actors use pressure power resources, only exploitation is likely to occur. However, where the difference in power resources between two actors is relatively small, the likelihood of success and the motivation of the weaker are high, something that increases the likelihood of overt conflicts. Although Korpi [38] did not mention female marriage migrants, the most important power resource in history for a disadvantaged population is human capital (e.g., labor power). Organizations play an especially important role in facilitating the mobilization of power resources and enabling collective action. Thus, there is an embedded different meaning in protest even if a single foreign wife civi who possess linking social capital. It is not just a single action of coping with stress but it could also be a collective action, which has the likelihood of overcoming discrimination and accessing useful resources [39].

Second, different distribution and health impact of discrimination experiences could, in part, be explained by the concept of empowerment and conscientization. Wallerstein [40] described empowerment as a social action process that motivates people to achieve goals of increasing political efficacy and social justice. A central strategy of empowerment is the process of conscientization, whereby people become aware of the political, socioeconomic and cultural contradictions that shape their lives and who they are [41]. It is argued that foreign wives who acquired the skills of critical reflexivity from the group of linking social capital are more likely to notice, recall, and report their experiences of racial discrimination. In contrast, foreign wives with non-linking social capital of class-segregated networks find it hard to create sufficient trust between different social groups and may be motivated to ignore their experiences of discrimination [42] or exaggerate experiences to avoid blaming themselves for failure [43]. Several studies have suggested that such internalization is related to chronic disease although it may have self-protective qualities under some circumstances [23, 24].

Third, although many variables of socioeconomic factors were inserted in the analytic model, the results of this study could not resolve the issue of endogeneity. Various determinants on health inequality neglected in this study (e.g., neighborhood concentration of immigrants, workplace safety, number of chronic disease, and prevalence of mental disorder) could be, in part, residual term. Szreter and Woolcock [5], who suggested the novel concept of linking social capital, stressed that there might be a crucial precondition more than shared language to create social capital between the haves and the have-nots. They indicated that ‘a shared sense of fairness’ is needed as a basis [5]. It might be the product of a prior history of political, constitutional, and ideological work on which to construct the conditions [39]. Although this concept seems more difficult to operationalize, it could also be included in the unexplained factors.

A major strength of this study is its large sample size from a nationally representative sample of South Korea. To the best of my knowledge, no studies have attempted to decompose discrimination-related health inequalities into their demographics and structural determinants related with immigration. Measuring health inequality using the variable of discrimination, not income, contains profound significance, especially in the research field of the immigrant’s health because economic deprivation might be not a unique determinant of health inequality among foreign immigrants. Economics is the discipline that has specialized in the analysis of exchange relationships under the assumption that every actor has a comparatively balanced power resource [38]. However, when if we consider the political status of foreign wives who did not possess the basic right of citizenship, analyzing income-related inequality is not sufficient to investigate health resilience factors. Furthermore, concerning the unequal distribution of income within families, the household income may not represent the living standard of foreign wives [44].

Despite the favorable outcomes and its theoretical implication, this study has several limitations. First, although the research design of this study helps identify group differences and factors that generate inequality, it is difficult to make causal inferences using a cross-sectional study. Second, limited predictive and explained power of the decomposition model were observed, as shown using the residual term, especially among the group linking social capital. Third, the potential problems of measurement are not well resolved in this study. A general concern with SRH is that they are based on respondent’s behaviors and understanding of their health status, which can make them prone to measurement error. The validity of the discrimination questionnaire (i.e., Experiences of Discrimination) for South Korean context is unknown. Additional studies are necessary to assess the cross-cultural and cross-language validation among foreign wives in South Korea. Also, there is lack of consistency or uniformity in the operationalization of linking social capital. Finally, the results should not be interpreted as though linking social capital may enhance the status of subjective health among foreign wives. In fact, the level of poor SRH and the participating group of linking social capital were negatively associated, if health effect is assessed by conditional-mean model (second low of Table 1). However, when we consider that migrants are typically healthier than their peers at origin and destination, which is likely because of selection bias (i.e., healthy migration effect and salmon effect), comparing the health status by conditional-mean model may be inappropriate at least in this cross-sectional design study. Attempting to reduce unacceptable and unjust health disparity, rather than enhancing the mean value of health status, may be a more appropriate goal considering the perspective of developing the migrant’s health policy.

## Conclusions

Despite these limitations, the present results indicated that linking social can be a useful resource of the health resilience factor that equalizes discrimination-related health inequality among marriage migrant women in South Korea. The theoretical plausibility was high considering the power resource theory and the known importance of the material aspect of social capital. Although this study did not reveal whether political participation or its preconditions is a critical factor, this study provided additional evidence to support that social capital must be placed in its proper political context.

## Abbreviations

CC: Concentration curve
CI: Concentration Index
SE: Standard error
SRH: Self-rated health
